# Metformin alters DNA methylation genome-wide via the H19/SAHH axis

**DOI:** 10.1038/onc.2016.391

**Published:** 2016-10-24

**Authors:** T Zhong, Y Men, L Lu, T Geng, J Zhou, A Mitsuhashi, M Shozu, N J Maihle, G G Carmichael, H S Taylor, Y Huang

**Affiliations:** 1 Department of Obstetrics, Gynecology, and Reproductive Sciences, Yale School of Medicine, New Haven, CT, USA; 2 Department of Laboratory Medicine, First Affiliated Hospital of Gannan Medical University, Ganzhou, Jiangxi, China; 3 Department of Head and Neck Surgery, State Key Laboratory of Oral Diseases, West China Hospital of Stomatology, Sichuan University, Chengdu, Sichuan, China; 4 Department of Chronic Diseases Epidemiology, Yale School of Public Health, Yale University School of Medicine, New Haven, CT, USA; 5 Department of Endocrinology, School of Medicine, First Affiliated Hospital of Xi'an Jiaotong University, Xi'an, Shaanxi, China; 6 Department of Surgical Oncology, Affiliated Sir Run Run Shaw Hospital, Zhejiang University School of Medicine, Hangzhou, Zhejiang, China; 7 Department of Reproductive Medicine, Graduate School of Medicine, Chiba University, Chiba, Japan; 8 Georgia Cancer Center, Medical College of Georgia, Augusta, GA, USA; 9 Department of Genetics and Genome Sciences, University of Connecticut Health Center, Farmington, CT, USA

## Abstract

The molecular mechanisms underlying the antineoplastic properties of metformin, a first-line drug for type 2 diabetes, remain elusive. Here we report that metformin induces genome-wide alterations in DNA methylation by modulating the activity of S-adenosylhomocysteine hydrolase (SAHH). Exposing cancer cells to metformin leads to hypermethylation of tumor-promoting pathway genes and concomitant inhibition of cell proliferation. Metformin acts by upregulating microRNA let-7 through AMPK activation, leading to degradation of H19 long noncoding RNA, which normally binds to and inactivates SAHH. H19 knockdown activates SAHH, enabling DNA methyltransferase 3B to methylate a subset of genes. This metformin-induced H19 repression and alteration of gene methylation are recapitulated in endometrial cancer tissue samples obtained from patients treated with antidiabetic doses of metformin. Our findings unveil a novel mechanism of action for the drug metformin with implications for the molecular basis of epigenetic dysregulation in cancer. This novel mechanism of action also may be occurring in normal cells.

## Introduction

Metformin, a biguanide compound, is among the most commonly used drugs worldwide for the treatment of type 2 diabetes owing to its high efficacy and minimal side effects. In recent years, there has been significant interest in metformin as a potential cancer chemopreventive and/or therapeutic agent (reviewed in Pollak^1^ and Pryor and Cabreiro^2^). Despite extensive research and ongoing multicentered randomized clinical trials on the efficacy of metformin as an anticancer agent, the antineoplastic mechanisms of action of this drug remain enigmatic. Both indirect (systemic) and direct mechanisms have been proposed (reviewed in Pollak,^1^ Pryor and Cabreiro,^2^ Foretz *et al.*,^3^ and Pavlova and Thompson^4^). By altering the endocrine-metabolic milieu of the host that is, reducing systemic levels of glucose and insulin), metformin may decrease cancer cell proliferation (reviewed in Pavlova and Thompson^4^). However, the magnitude of these systemic effects may not be sufficient to impact cancer cell growth directly, especially in patients with normal baseline glucose and insulin levels or in cancers that are insensitive to insulin. Alternative systemic effects of metformin have, therefore, been proposed, including inhibition of pro-inflammatory cytokines and augmentation of host immune response to cancer cells. In contrast, dozens of other studies using *in vitro* and/or rodent models suggest that metformin may directly interact with cancer cells to elicit its antineoplastic effect. Favored mechanisms, to date, include induction of ‘energy stress' (through inhibition of the mitochondrial respiratory chain complex I) and activation of the key energy sensor AMPK and subsequent mTOR inhibition, thereby leading to tumor suppression. While alterations in cancer cell metabolism through both AMPK-dependent and AMPK-independent pathways contribute to the direct cellular effects of metformin, the precise mechanisms leading to these metabolic changes have yet to be defined. In addition, it remains to be determined whether the effects of metformin observed *in vitro* and in animal models actually occur in human tissues and in particular, whether conventional antidiabetic doses of metformin are adequate to achieve these effects, in part, because most preclinical studies to date have used drug levels significantly higher than those used in patients with diabetes. Adequately addressing these questions will be key to the success of ‘repurposing' metformin for its potential use as a cancer chemopreventive or therapeutic agent.

We previously reported that metformin reduces the motility and invasiveness of both endometrial and ovarian cancer cells in part by decreasing the production of H19.^5^ H19 is a long noncoding RNA that is highly expressed during fetal development but strongly downregulated in most adult tissues. Interestingly, however, H19 is aberrantly expressed in almost all cancer types tested, where it has been shown to play an important role in tumor biology (reviewed in Matouk *et al.*^6^ and Raveh *et al.*^7^). We recently found in muscle cells (murine) that H19 alters DNA methylation genome-wide through its interaction with S-adenosylhomocysteine hydrolase (SAHH), the only eukaryotic enzyme capable of hydrolyzing S-adenosylhomocysteine (SAH), a strong feedback inhibitor of SAM-dependent methyltransferases including DNA methyltransferases (DNMTs). H19 binds to SAHH and inhibits its hydrolytic activity.^8^ We showed that in mouse skeletal muscle cells decreasing H19 by siRNA activates SAHH and facilitates the removal of SAH, which in turn relieves the inhibition of DNMT3B by SAH and enables it to methylate a subset of genes.^8^ Exposing human endometrial or ovarian cancer cells to metformin decreases H19 levels (although how metformin decreases H19 remains to be investigated), with a concomitant increase in methylation within the promoter region of *H19*.^5^ Whether this methylation change is mechanistically linked to metformin's action in cancer cells or is just coincidental remains to be investigated. These findings raise the intriguing possibility that metformin may function to alter gene methylation in malignant cells.

Here, we show that metformin induces let-7-mediated H19 degradation through activation of AMPK. Exposing endometrial cancer cells to metformin leads to global DNA methylation changes mediated through the H19/SAHH axis. Impressively, these same metformin-induced H19 repression and gene methylation changes are also observed in human tumor samples derived from endometrial cancer patients treated with metformin. These studies provide further support for the notion that the dose range of metformin commonly used for the treatment of diabetes has the potential to directly impact cancer cell biology through an AMPK-dependent mechanism.

## Results

### Metformin increases *H19* methylation through the H19/SAHH axis

**Figure 1:**
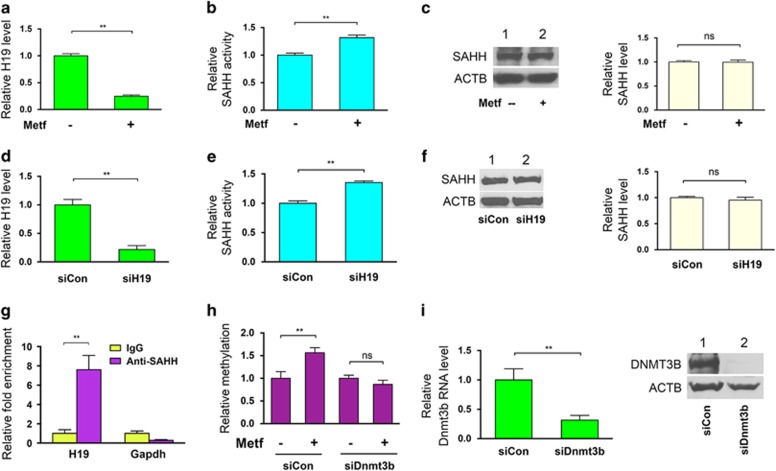
Metformin-induced H19 reduction activates SAHH leading to DNMT3B-dependent hypermethylation of *H19* promoter. (**a**–**c**) ARK2 cells were treated with (+) or without (−) metformin (metf) at a final concentration of 2 mm for 48 h. H19 RNA levels (**a**), *in vivo* SAHH activity (**b**) and SAHH protein levels (**c**) were assessed by reverse transcriptase (RT)–quantitative PCR (qPCR), SAHH activity assay and western blot analysis, respectively. In (**c**), representative gel images of three independent western blot experiments are shown, with quantification on the right. (**d**–**f**) ARK2 cells were transfected with control siRNA (siCon) or H19-specific siRNA (siH19). H19 RNA levels (**d**), *in vivo* SAHH activity (**e**) and SAHH protein levels (**f**) were assessed. (**g**) RNA immunoprecipitation (RIP) with mouse monoclonal anti-SAHH antibody or preimmune IgGs from ARK2 cells. RNA levels in immunoprecipitates were determined using RT–qPCR. Levels of H19 and GAPDH mRNA are presented as fold enrichment in anti-SAHH relative to IgG immunoprecipitates. (**h**) ARK2 cells were transfected with siCon or Dnmt3b-specific siRNA (siDnmt3b). Twenty-four hours later, cells were treated with or without metformin, followed by QMSP analysis of *H19* promoter methylation 24 h later. (**i**) Confirmation of Dnmt3b knockdown by RT–qPCR (left panel) and western blot analysis (right panel). Numbers are mean±s.d. (*n*=3). **P<0.01. NS, no statistical difference.

We previously observed that treatment of the endometrial cancer-derived cell line ARK2 with the biguanide antidiabetes drug metformin results in a decrease in H19 levels and an increase in methylation within the promoter region of *H19*.^5^ Whether these changes were mediated by the H19/SAHH axis, however, as previously had reported in normal mouse muscle cells^8^ was unclear. To address this possibility, we first asked whether downregulation of H19 following metformin exposure would activate SAHH. ARK2 cells were incubated with metformin followed by measurement of H19 levels and SAHH activity 24 h later. Metformin treatment reduced H19 levels by ~4-fold (Figure 1a), and increased SAHH activity by ~20% (Figure 1b) without affecting its protein level (Figure 1c). Importantly, knockdown of H19 expression using siH19^5^ to levels comparable to those induced by metformin(Figure 1d, compared with Figure 1a) also increased SAHH activity by ~20% (Figure 1e) without altering the level of this protein (Figure 1f). Moreover, the physical interaction between SAHH and H19, which previously had been observed in mouse muscle cells,^8^ also was observed in ARK2 cells by RNA immunoprecipitation. Using an SAHH-specific antibody (Anti-^SAHH8^), we observed an ~8-fold enrichment of H19 in SAHH-containing ribonucleoprotein complexes (RNPs) relative to control immunoglobulin G (IgG) immunoprecipitates (Figure 1g, left column), whereas a GAPDH mRNA (control) was not enriched under these circumstances (right column). Together, these results support the hypothesis that metformin-induced downregulation of H19 activates SAHH in human endometrial cancer cells.

Changes in SAHH activity change could potentially affect all DNMTs, including DNMT1, DNMT3A and DNMT3B. Here we focused initial studies on DNMT3B, because it previously has been shown to be associated with the H19/SAHH pathway.^8^ To determine whether DNMT3B may contribute to the observed metformin-induced *H19* promoter methylation, DNMT3B was downregulated in ARK2 cells using siDnmt3b,^9^ and the effects on *H19* promoter methylation were evaluated using quantitative methylation-specific PCR (QMSP).^8^ While metformin increased *H19* promoter methylation (Figure 1h, compare second bar with first bar on left) as previously reported,^5^ the combination of this drug with Dnmt3b knockdown (Figure 1i) restored the level of *H19* promoter methylation to that of the control (Figure 1h, third and fourth bars on right). These results suggest that DNMT3B may contribute to metformin-induced *H19* hypermethylation, although possible contributions from other DNMTs cannot be excluded at this time.

### Let-7 mediates metformin-induced H19 destabilization

**Figure 2:**
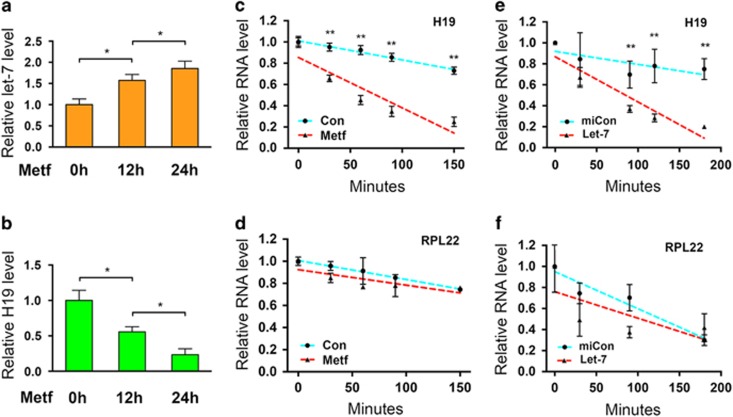
Metformin increases let-7 production, which targets H19 for destabilization. (**a**, **b**) ARK2 cells were treated with metformin, followed by RNA extraction and reverse transcriptase (RT)–quantitative PCR (qPCR) to determine RNA levels of let-7 (**a**) and H19 (**b**). (**c**, **d**) ARK2 cells were treated with metformin or water control (Con) for 2 h. Transcription inhibitor actinomycin D was added at a final concentration of 5 μg/ml, followed by RNA extraction at the indicated time points. RNA levels of H19 (**c**) and RPL22 (**d**, as a negative control) were measured by RT–qPCR. (**e**, **f**) ARK2 cells were transfected with let-7 mimic (Let-7) or negative control microRNA (miCon) in the presence of actinomycin D. H19 (**e**) and RPL22 (**f**) RNA levels were determined by RT–qPCR. Numbers are mean±s.d. (*n*=3). **P<0.01. *P<0.05.

Our previous studies from mouse muscle cells revealed a double negative-feedback loop between H19 and let-7 in which H19 blocks let-7 function by sequestering let-7, while binding of let-7 to H19 triggers H19 degradation.^10, 11^ Since metformin decreases the steady-state level of H19 in ARK2 cells (Figure 1a and see Yan *et al.*^5^), we anticipated that metformin also might induce H19 degradation by upregulating let-7. In fact, exposing the human breast cancer cell line MCF-7 to metformin has been reported to increase let-7 levels, but the underlying mechanism of this metformin effect has never been determined.^12^ To test whether metformin might effect let-7 levels in endometrial cancer cells, ARK2 cells were incubated with metformin, followed by measurement of both let-7 and H19 RNA levels at 12 and 24 h. As illustrated in Figure 2, the increase in let-7 levels (Figure 2a) coincides with a decrease in H19 levels (Figure 2b) at both time points tested. RNA stability analyses revealed an accelerated degradation of H19 (Figure 2c) but not of RPL22 (ribosomal protein L22; control) mRNA (Figure 2d) in the presence of metformin. In addition, transfection of a let-7 mimic into ARK2 also destabilized H19 (Figure 2e) but not RPL22 mRNA (Figure 2f). Further, transfection of a let-7 inhibitor iLet-7^5^ abolished metformin-induced H19 reduction (Supplementary Figure S1). Together, these results suggest that metformin upregulates let-7 in endometrial cancer cells, resulting in H19 degradation.

### Let-7 upregulation involves AMPK activation

**Figure 3:**
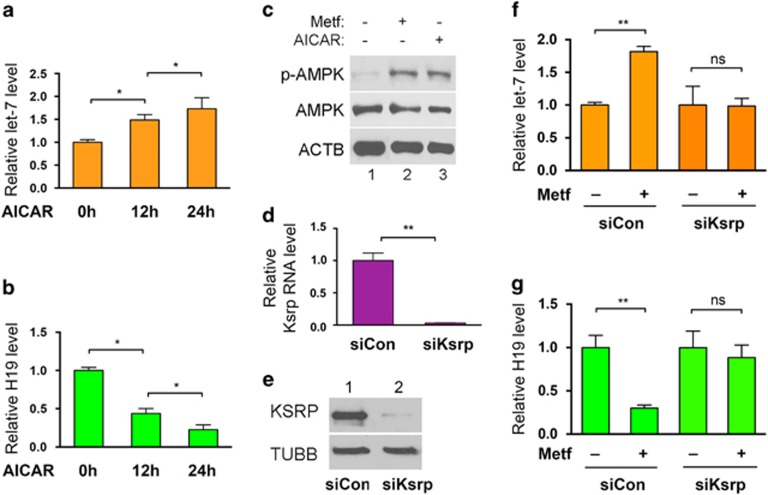
AMPK activation by metformin increases the biogenesis of let-7, which requires KSRP. (**a**, **b**) ARK2 cells were treated with AICAR (final concentration 0.5 mm), followed by RNA extraction and reverse transcriptase (RT)–quantitative PCR (qPCR) to determine RNA levels of let-7 (**a**) and H19 (**b**). (**c**) ARK2 cells were treated with or without metformin or AICAR, followed by western blot analysis for phosphorylated AMPK (p-AMPK) and total AMPK (AMPK) using β-actin (ACTB) as a loading control. Representative western blot gels of three independent experiments are shown. (**d**, **e**) ARK2 cells were transfected with siCon or Ksrp-specific siRNA (siKsrp), followed by RT–qPCR (**d**) and western blot analysis (**e**) to confirm KSRP knockdown at the mRNA and protein levels, respectively. (**f**, **g**) ARK2 cells were transfected with siCon or siKsrp, followed by treatment with or without metformin 24 h later. RNA levels of let-7 (**f**) and H19 (**g**) were determined by RT–qPCR 24 h post-transfection. Numbers are mean±s.d. (*n*=3). **P<0.01. *P<0.05. NS, no statistical difference.

Metformin exerts its anticancer effects, in part, by activating AMPK.^1, 2, 3^ Indeed, we previously have reported increased phosphorylation of AMPK in patient tumor samples taken from patients participating in a preoperative prospective clinical trial. The endometrial cancer patients participating in this trial were treated with 1500–2250 mg/day, which is in the range of metformin used to treat diabetes patients.^13^ To test whether AMPK activation might contribute to let-7 upregulation *in vitro*, ARK2 cells were incubated with 5-aminoimidazole-4-carboxamide riboside (AICAR), an AMPK-activating agent,^14^ followed by measurement of let-7 and H19 RNA levels. AICAR treatment led to an increase in let-7 RNA levels at both the 12 and 24 h time points (Figure 3a), with a concomitant decrease in H19 levels (Figure 3b). As expected, treatment of ARK2 cells with metformin or with AICAR increased AMPK phosphorylation at Thr172, consistent with AMPK activation (Figure 3c).^13, 15^ Together, these results support the hypothesis that AMPK activation by metformin contributes to let-7 upregulation in endometrial cancer cells.

KSRP is a key factor in promoting processing of let-7 precursors to mature let-7.^16^ To test whether KSRP also might play a role in metformin-induced let-7 upregulation, KSRP knockdown experiments using a KSRP-specific siRNA (siKsrp^17^) were conducted in the presence or absence of metformin, followed by measurement of let-7 and H19 RNA levels in ARK2 cells. siKsrp treatment reduced KSRP at both the mRNA (Figure 3d) and protein (Figure 3e) levels. In control siRNA-transfected cells, metformin increased let-7 (Figure 3f, left two columns) and decreased H19 RNA levels (Figure 3g, left two columns). These effects were lost when KSRP was downregulated (Figures 3f and g, right two columns), suggesting that KSRP is required for metformin-induced let-7 upregulation and subsequent H19 destabilization.

### The H19/SAHH/DNMT3B pathway mediates metformin effects in other cancer cells

**Figure 4:**
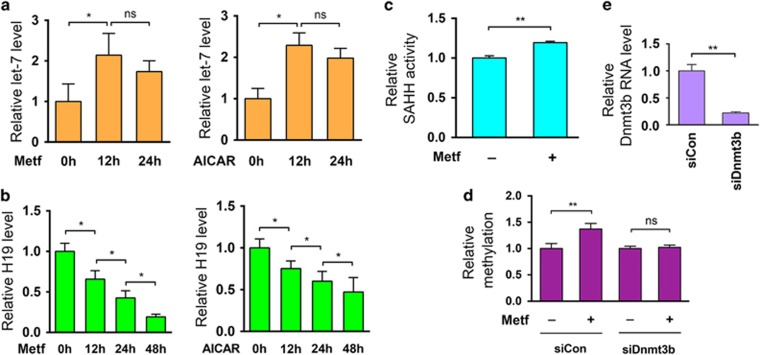
Metformin-induced let-7 upregulation and H19 repression activates SAHH, leading to DNMT3B-mediated hypermethylation of *H19* promoter in breast cancer MCF-7 cells. (**a**, **b**) MCF-7 cells were treated with metformin or AICAR for the indicated time frame, followed by measurement of RNA levels of let-7 (**a**) and H19 (**b**) 12 and 48 h later. (**c**) MCF-7 cells were treated with or without metformin, followed by evaluation of *in vivo* SAHH activity. (**d**) MCF-7 cells were transfected with siCon or siDnmt3b. Twenty-four hours later, cells were treated with or without metformin, followed by QMSP analysis of *H19* promoter 24 h later. (**e**) reverse transcriptase–quantitative PCR analysis to confirm Dnmt3b knockdown 48 h following siRNA transfection in MCF-7 cells. Numbers are mean±s.d. (*n*=3). **P<0.01; *P<0.05; NS, no statistical difference.

To address the possibility that the H19/SAHH/DNMT3B pathway mediates metformin effects in other cancer cells, we tested the effects of metformin on the human breast cancer-derived cell line MCF-7. As mentioned above, previous studies have shown that metformin treatment results in an increase in let-7 levels in breast cancer-derived MCF-7 cells.^12^ Here we confirmed and extended these results by demonstrating that metformin (or AICAR) treatment leads to an increase in let-7 (Figure 4a), a decrease in H19 (Figure 4b) and an increase in SAHH activity (Figure 4c) in MCF-7 cells. Metformin treatment also results in increased *H19* promoter methylation in MCF-7 cells (Figure 4d, left two columns), and this increase could be abrogated (Figure 4d, right two columns) by repression of DNMT3B expression using siDnmt3b (Figure 4e). Similar results were observed in human liver cancer cell lines (data not shown). Together, these results suggest that the H19/SAHH/DNMT3B axis is active in multiple cancer types, where it can mediate metformin-induced hypermethylation of *H19*.

### Metformin alters DNA methylation genome-wide

Given that siRNA-mediated H19 knockdown alters DNA methylation genome-wide in mouse muscle cells,^8^ and that metformin downregulates H19 (Figure 1a),^5^ we sought to determine whether exposure of endometrial cancer cells to metformin would induce changes in global DNA methylation. Thus, ARK2 cells were incubated with metformin for 48 h, followed by genome-scale DNA methylation profiling using previously described methods.^8^ Metformin treatment led to extensive methylation changes genome-wide compared with control-treated cells (Supplementary Figure S2 and Supplementary Data S1, and Gene Expression Omnibus (GEO) accession number GSE85974). Some genes became hypermethylated, others became hypomethylated and a third group showed no significant change in methylation. These observations were reminiscent of our previous findings studying siRNA-mediated H19 downregulation in mouse muscle cells.^8^ The differential effects of metformin on gene methylation were not surprising since these changes likely reflect a composite of both the direct and indirect effects of metformin. For example, activation of SAHH as a result of H19 repression by metformin would facilitate not only DNA methylation by DNMTs (direct effects) but also methylation of proteins that regulate chromatin structures (and DNMT accessibility), which in turn could influence DNA methylation (indirect effects). Indeed, the methylation status of a given gene is determined not only by activation of DNMTs but also by modifications of chromatin-bound proteins.^18, 19, 20^

**Figure 5:**
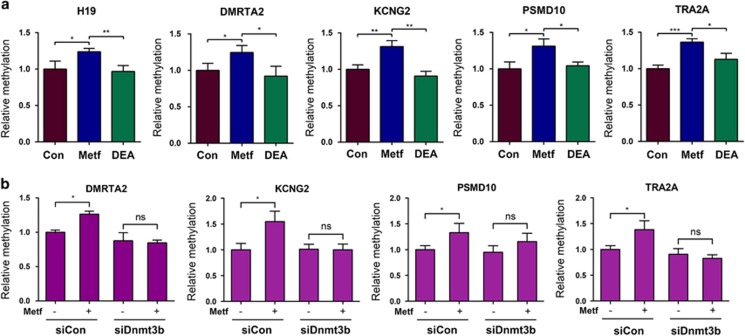
SAHH and DNMT3B are required for metformin-induced methylation changes in the DMRs of the indicated genes. (**a**) ARK2 cells were treated with control (Con), metformin (Metf) or metformin plus inhibitor (DEA). DNAs were isolated 48 h later and analyzed by QMSP using primers specific for the individual DMR. (**b**) ARK2 cells were transfected with siCon or siDnmt3b. Twenty-four hours later, cells were treated with or without metformin, followed by DNA extraction and QMSP analysis of the indicated DMRs 24 h later. Numbers are mean±s.d. (*n*=3). **P<0.01. *P<0.05. NS, no statistical difference.

Thus, it is reasonable to predict that a subset of the genes that become hypermethylated following metformin treatment may result from the direct effect of metformin. To test this hypothesis we randomly selected four genes from the top 2000 gene list showing strong hypermethylation in the promoter region to test as proof of principle (in addition to *H19*) following metformin treatment (Supplementary Data S1). Among these genes, *DMRTA2* encodes a transcription factor involved in human female germ cell development;^21^ *KCNG2* encodes a potassium channel subunit whose altered expression has been associated with malignant glial tumors;^22^ and *PSMD10* encodes a regulatory subunit of the 26S proteasome. Constitutive activation of this gene contributes to hepatocarcinogenesis.^23^ *TRA2A* encodes an RNA binding protein involved in the regulation of pre-mRNA splicing. Its increased expression has been found in hepatocellular carcinoma.^24^ ARK2 cells were again incubated with metformin (or control) in the presence or absence of the pharmacological SAHH inhibitor DEA.^8^ QMSP analysis was performed 48 h later using primers that specifically amplify a single differentially methylated region (DMR) of the genes chosen for further study. Metformin increased methylation in all five genes tested (Figure 5a, compare blue bars with brown bars). However, the combination of metformin with DEA restored the methylation of all five genes to control levels (compare green bars with blue bars), consistent with metformin-induced, SAHH-dependent regulation of methylation at these DMRs. To address whether DNMT3B was the downstream effector of this observed regulation (Figure 4d), DNMT3B was downregulated by siDnmt3b and the effects on methylation in the presence or absence of metformin treatment were examined. While metformin predictably increased methylation (Figure 5b, compare second bars with first bars, on left), its combination with DNMT3B knockdown restored methylation levels to control levels (Figure 5b, compare third bars with fourth bars), providing evidence to support the involvement of DNMT3B in this regulatory pathway. Taken together, these results are consistent with the notion that metformin can induce methylation changes at numerous genomic loci and that a subset of these changes is mediated through metformin's effects on the H19/SAHH/DNMT3B pathway.

### Metformin induces hypermethylation of tumor-promoting pathway genes

To provide biological insight into the observed metformin-induced methylation changes, we performed Ingenuity Pathway Analysis (IPA; see Albitar *et al.*^25^). Based on the common but admittedly oversimplified assumption that hypermethylation is associated with gene repression (and hypomethylation with gene activation), we stratified genes that showed significant methylation changes (*P*-value <0.05) into three categories depending on the location of methylation: promoter only (category 1), gene body only (category 2), and both promoter and gene body (category 3). While no activation or inhibition of genes in categories 1 or 3 were found to be statistically significant (based on a cutoff of *z*-score of <−2 being inhibition and >2 being activation), a subset of tumor-promoting pathway genes was inactivated in category 2. These included those genes encoding proteins involved in the development of cancers of epithelial tissues (Supplementary Data S2, red highlighted). Interestingly, in the same category, genes involved in neuronal development and function, cell morphology, and intercellular communications also were observed to be activated.

As many cancer genes function to promote cell proliferation and/or survival, we tested whether metformin treatment might negatively effect these biological end points. Thus, ARK2 and MCF-7 cells were exposed to metformin for 48 h, followed by cell viability and caspase-3/7 activity assays. Metformin treatment reduced cell viability without affecting caspase activity in both cell types (Supplementary Figure S3), consistent with the role of this pathway in regulating cell proliferation but not cell survival.

### Metformin-induced H19 repression and associated gene methylation changes are recapitulated in patient tumor samples

**Figure 6:**
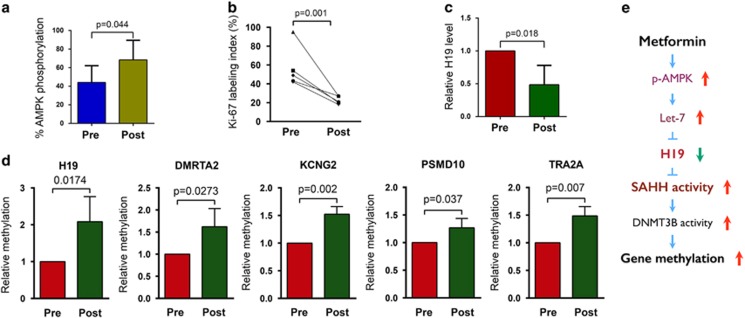
Metformin-induced H19 repression and alteration of gene methylation are recapitulated in endometrial cancer patients treated with antidiabetic doses of metformin. (**a**) Results of western blot analysis of AMPK phosphorylation in five paired specimens from before (Pre) and after (Post) metformin treatment. *P-*value is shown on top of the bar graph. (**b**) Results of quantification of immunostaining of Ki-67 in five paired endometrial cancer tissues. (**c**) Results of reverse transcriptase–quantitative PCR analysis of H19 RNA levels in five paired endometrial cancer tissues. (**d**) Results of QMSP of methylation of the indicated genes in four paired endometrial cancer tissues. *P*-values are shown on top of each bar graph. (**e**) A model for metformin-induced, H19-mediated alteration of gene methylation in cancer cells.

We previously reported that preoperative treatment of patients with endometrial cancer using antidiabetic doses of metformin decreased tumor cell proliferation with a concomitant increase in AMPK activation.^13^ Here we wanted to test whether similar levels of metformin treatment in patients with endometrial cancer would also result in changes in H19 expression and/or alterations in gene methylation. Thus, metformin was administered preoperatively to five endometrial cancer patients (see Supplementary Table S1 for patient characteristics) for 3–12 weeks. AMPK phosphorylation, cell proliferation, H19 levels and DNA methylation were measured in tumor tissue samples from both pre- and postoperative samples. As previously reported,^13^ preoperative metformin treatment resulted in an increase in AMPK phosphorylation (Figure 6a) as well as decreased tumor cell proliferation (Figure 6b). Strikingly, metformin treatment also resulted in decreased H19 levels (Figure 6c) and increased methylation in each of the five genes (Figure 6d) previously tested *in vitro* (see Figure 5a).

## Discussion

Using human cancer cell lines as a model system, we show that metformin functions to alter DNA methylation in a genome-wide fashion and that this action is at least in part mediated by the H19/SAHH/DNMT3B axis. As illustrated in Figure 6e, exposing cells to metformin activates AMPK leading to increased levels of let-7, which in turn targets H19 for degradation. This frees SAHH from sequestration by H19, which would otherwise prevent it from hydrolyzing SAH, a feedback inhibitor of DNMT3B, with an end point of increased DNA methylation. The biological significance of these results is highlighted by the observation of similar findings in parallel studies using human endometrial cancer tissue samples from patients treated with doses of metformin commonly used for the treatment of diabetes. Together, these studies provide strong support for a direct role of metformin in regulation of those gene expression events that play a vital role in aspects of cancer cell biology. Given that this regulatory pathway also appears to function in other cancer cell types (Figure 4 and data not shown), and that diverse types of cancer cells express H19 (reviewed in Matouk *et al.*^6^ and Raveh *et al.*^7^), our findings support the conclusion which have proposed that metformin warrants further clinical investigation as both a chemopreventative agent and as a potential therapeutic agent in certain cancer patients.

Moreover, our studies establish an important mechanistic link between metformin and *H19* promoter hypermethylation. It is intriguing to speculate that H19 degradation as a result of AMPK activation and let-7 upregulation induced by metforminmay serve as an initial trigger that ultimately leads to chronic inhibition of *H19* transcription due to promoter hypermethylation. Such a feedforward mechanism would help to reinforce metformin's action on H19 repression in cancer cells.

These findings also identify a new mechanism of epigenetic dysregulation in cancer cells. Many mechanisms have been proposed to explain H19's role in tumor initiation and progression, including inhibition of the tumor suppressor retinoblastoma *Rb* through H19-encoded miR-675;^26, 27^ alteration of gene transcription via interaction with Polycomb repressive complex 2 components;^28^ and sequestration and inhibition of the tumor suppressor microRNA let-7.^5, 11^ Our studies suggest that another mechanism of H19-mediated carcinogenesis may also be at play: genome-wide dysregulation of gene methylation. Since the initial discovery of methylation changes in specific genes in primary human tumors,^29^ epigenetic dysregulation has been increasingly recognized as a hallmark of cancer^30, 31^ (reviewed in Sandoval and Esteller^32^). Three types of alterations in DNA methylation have been documented in cancers: hypermethylation, hypomethylation and loss of imprinting. Epigenetic alterations in tumors also include histone modifications and H19-induced SAHH activity changes which itself could alter histone modification. We propose here that H19, in conjunction with SAHH, may contribute to alterations in the epigenetic landscape in cancer cells. Similar to other hallmarks of cancer, H19 may therefore also serve as a useful biomarker for such clinical applications as predicting cancer recurrence, metastasis, or even as a potential marker predicting therapeutic response, since DNA methylation-based biomarkers have found increasing utility in the clinic in recent years (reviewed in Sandoval and Esteller^32^).

While the proposal that metformin induces hypermethylation of certain tumor-promoting pathway genes (Supplementary Table S2) is consistent with metformin's proposed anticancer mechanism of action, we acknowledge that this interpretation is based on the assumption that hypermethylation will result in inhibition of gene expression. In fact, the relationship between methylation and gene expression is far more complex; not only is it genomic context-dependent, it is also influenced (positively and negatively) by factors such as distance between CpGs relative to transcription start-sites and whether these sites are localized in promoters, enhancers or gene bodies. Adding an additional layer of complexity, the patterns and types of modifications to chromatin-bound proteins also can affect gene expression.^18, 19, 20^ We further acknowledge that the changes we have observed in SAHH activity could potentially affect many SAM-dependent methyltransferases that methylate other cellular components (for example, methylated proteins). We, therefore, propose here that it is ultimately the net affect of metformin's influence on gene expression, mediated at least in part via DNA methylation, that results in its anticancer potential.

Based on these results, we propose that metformin warrants further study as a potential anticancer agent, including the mechanisms, such as regulation of gene expression, that may contribute to this anticancer activity. In this regard, metformin has been widely prescribed not only for the treatment of diabetes (type 2) but also for the prevention of diabetes in patients with insulin resistance. The patients included in our study were not diabetic but did have insulin resistance/glucose intolerance. There is, therefore, precedent for the safe use of metformin in endometrial cancer patients (for the prevention of diabetes), as well as an extensive history of using this drug in humans (for diabetes), suggesting that the risk profile associated with this drug, as part of future testing as an anticancer agent, should be acceptable. Therefore, metformin is potentially useful in the prevention of diabetes in endometrial cancer patients.

In conclusion, our studies extend the known mechanisms by which metformin can exert its effects on gene expression in cancer cells, and demonstrate for the first time that metformin is able to directly impact cancer cell proliferation by altering DNA methylation via regulation of the H19/SAHH axis. These studies further suggest that this mode of action may contribute to the observed and predicted anticancer activities of metformin *in vivo*. Future studies aimed at developing a better understanding of the molecular interplay between various epigenetic factors will be required to more fully appreciate the specific role of metformin-induced epigenetic modifications in specific cancers, such as endometrial and breast cancer, and such studies should be complemented through the conduct of ongoing clinical trials of metformin as a potential chemopreventative agent in endometrial cancer—particularly in high-risk populations (https://clinicaltrials.gov/ct2/show/NCT01697566). It remains to be determined whether this newly identified metformin mechanism also exists in non-cancerous cells.

## Materials and methods

### Antibodies, siRNAs, miRNAs, activators and inhibitors

Antibodies for SAHH (for RNA immunoprecipitation, Santa Cruz, Dallas, TX, USA; sc-271389; for western blot, Proteintech Group, Chicago, IL, USA; 10757-2-P), DNMT3B (Novus, Littleton, CO, USA; NB300-516), KSRP (Cell Signaling, Boston, MA, USA; 13398), Phospho-AMPK-alpha (Thr172) (Cell Signaling; 2535), AMPK alpha (Cell Signaling; 2532), β-tubulin (Abcam, Cambridge, MA, USA; ab6046), β-actin (Abcam; ab8226) and mouse preimmune IgGs (Chemicon, Billerica, MA, USA; PP54) were purchased. Control siRNA (siCon; Ambion, Grand Island, NY, USA; AM4636), siRNAs specific for human H19 (siH19; Ambion; 4390771/n272452), KSRP (siKsrp; Ambion; 4390824/s16322) and DNMT3B (siDnmt3b; Santa Cruz; SC-37759), and let-7a mimics (Let-7; Ambion; AM17100/PM10050), Pre-miR negative control (miCon; Ambion; AM17110), Let-7 inhibitor (iLet-7; Ambion; 4392431) and miRNA control (Ambion; AM17010) were purchased. Metformin (ALX-270-432-G005), AICAR (123040) and D-Eritadenine (sc-207632) were from ENZO Life Sciences International Inc. (Uniondale, NY, USA) Calbiochem (Billerica, MA, USA), and Santa Cruz, respectively. Metformin and AICAR were used at final concentrations of 2 and 0.5 mm, respectively.

### Cell culture and siRNA/iLet-7 transfection

The ARK2 and MCF-7 cells were authenticated and were free from mycoplasma contamination. The cells were cultured in RPMI1640 (Gibco, Grand Island, NY, USA; 11965-092) supplemented with 10% fetal bovine serum, heat inactivated, 1% amphotericin B, 1% penicillin/streptomycin and 1% L-glutamine. Cells were transfected in a 48-well plate scale. To prepare siRNA transfection solution for each well, 16 pmol of siCon or siH19 was mixed with 50 μl OPTI-MEM by gentle pipetting. In parallel, 0.5 μl Lipofectamine 2000 was mixed with 50 μl OPTI-MEM. Following 5 min of incubation at room temperature, the two were mixed by gentle pipetting and incubated for 20–30 min at room temperature to allow siRNA/lipid complexes to form. At the end of incubation, the 100 μl transfection solution was used to re-suspend cell pellet (4 × 10^4^ cells). After incubation at room temperature for 10 min, regular growth medium was added at a ratio of 1:5 (1 volume of transfection solution/5 volumes of growth medium) and the cell suspension was transferred to the culture plate. After 12 h incubation at 37 °C in 5% CO_2_, the medium was replaced with fresh growth medium. RNAs and proteins were extracted and analyzed at the indicated time points following transfection. For metformin/iLet-7 experiments, 45 pmol of iLet-7 or control inhibitor were used for each well of cells.

### *In vivo* SAHH activity assay

The experiments were performed in a 96-well scale using the Human Homocysteine (Hcy) ELISA Kit (Mybiosource, San Diego, CA, USA; MBS260128) that allows quantitative measurement of homocysteine concentration in cell extracts, according to the manufacturer's instructions. The concentration of homocysteine in cell extracts was used as a readout for *in vivo* SAHH activity,^8^ as SAHH hydrolyzes SAH to homocysteine and adenosine. Briefly, ARK2 cells were washed with cold phosphate-buffered saline and lysed on plate in 200 μl of lysis buffer (40 mm hexadecyltrimethylammonium bromide, 75 mm Tris-HCl, pH 8.0, 1m NaCl, 15 mm EDTA). The lysate was cleared of insoluble materials by centrifugation at 15 000 *g* at 4 °C for 15 min. Immediately following the centrifugation, 100 μl of the supernatant was collected and used for SAHH activity measurement. The absorbance of the samples was determined using a FilterMax F3&F5 Multi-Mode Microplate Reader (Molecular Devices).

### RNA extraction and reverse transcriptase–quantitative PCR

Total RNAs were extracted from cells using PureLink RNA Mini Kit (Ambion; catalog number 12183025). cDNA was synthesized using PrimeScript RT Reagent Kit (Takara, Mountain View, CA, USA; RR037A) in a 20 μl reaction containing 100–500 ng of total RNA. Real-time quantitative PCR (qPCR) was performed in a 15 μl reaction containing 200–800 ng of total RNA. PCR was performed by initial denaturation at 95 °C for 5 min, followed by 40 cycles of 30 s at 95 °C, 30 s at 60 °C and 30 s at 72 °C. Specificity was verified by melting curve analysis and agarose gel electrophoresis. The threshold cycle (Ct) values of each sample were used in the post-PCR data analysis. Gene expression levels were normalized against β-tubulin. Real-time PCR primers are listed in Supplementary Table S2.

### Let-7 miRNA quantification

Total RNAs were extracted from ARK2 cells using the PureLink RNA Mini Kit. Levels of mature let-7 were determined by reverse transcriptase–qPCR using miScript reverse transcription kit (catalog number 218161) and miScript SYBR Green PCR kit (catalog number 218073) according to the manufacturers' instructions. PCR primer sets (miScript primer) specific for let-7a (MS00006482) and snRNA U6 (MS00033740) were purchased from Qiagen (Hilden, Germany). The indicated miRNA levels were normalized against U6.

### RNA immunoprecipitation

To prepare antibodies, 20 μl of protein A Sepharose beads were incubated with 20 μg of monoclonal anti-SAHH antibody or 20 μg of mouse preimmune IgG in 500 μl IP buffer (0.5% Triton X-100, 200 mm NaCl, 10 mm Tris-HCl at pH 7.5 and 10 mm EDTA) at 4 °C overnight. The next day, the beads were washed three times with IP buffer and kept on ice until used. To prepare cell lysates, ARK2 cells (from 1 well of a 6-well plate) were harvested and cell pellets resuspended in 600 μl of of freshly prepared lysis buffer (0.5% Triton X-100, 10 mm NaCl, 10 mm Tris-HCl at pH 7.5, 10 mm EDTA, 0.5 mm PMSF, 1 mm DTT, 1 × protease inhibitor cocktail (Calbiochem) and 400 units/ml RNase inhibitor). The suspensions were incubated on ice for 20 min. After removing insoluble materials by centrifugation, lysates were precleared using 10 μl of protein A sepharose (NaCl was added to a final concentration of 200 mm), followed by addition of yeast tRNA (Ambion) to a final concentration of 40 μg/ml. The cleared lysates were transferred to tubes containing antibody or preimmune IgG-coated beads, and IP was carried out by rotating the tubes at 4 °C for 3 h. Following IP, the beads were washed five times with IP buffer by adding 1 ml of the buffer and rotating the tube at 4 °C for 2 min each time. RNA was extracted from the beads using PureLink RNA Mini Kit (Ambion; catalog number 12183018A). Reverse transcription was performed in a 40 μl reaction volume using the Bio-Rad iScript cDNA synthesis kit, followed by qPCR.

### RNA stability analysis

To evaluate let-7 effects on H19 RNA stability, miRNA transfection (48-well plate scale) combined with actinomycin D time course analysis was performed. To prepare transfection cocktail, 1 pmol of control miRNA (miCon) or let-7a mimic was mixed with 50 μl of OPTI-MEM. In parallel, 0.5 μl of Lipofectamine 2000 was mixed with 50 μl of OPTI-MEM. Following 5 min of incubation, the two solutions were mixed and incubated at room temperature for 20 min. The resulting 100 μl of transfection cocktail was added to ARK2 cells pre-washed with OPTI-MEM. Upon adding the transfection cocktail, actinomycin D was also added to each well at a final concentration of 10 μg/ml. Total RNA was extracted at the indicated time points, followed by reverse transcriptase–qPCR analysis. Results are presented after normalization against β-tubulin mRNA levels with 0 time point RNA levels arbitrarily set as 1.

### Western blot analysis

Cell pellets were quickly lysed in five volumes of 2 × sodium dodecyl sulfate sample buffer heated at 95 °C for 5 min, with occasional vortexing. Five to 10 μl of homogenized samples were loaded onto 10% SDS gel, followed by western blot analysis. The linear dynamic range of each protein of interest was determined by serial dilutions. Bands on western blot gels were quantified using Image J.

### Genomic DNA extraction

Genomic DNA was isolated using Quick-gDNA MicroPrep (Zymo, Irvine, CA, USA; D3021) according to the manufacturer's instructions.

### Quantitative methylation-specific PCR (QMSP)

Genomic DNA was extracted from ARK2 and MCF-7 cells in one well of six-well plates using Quick-gDNA MicroPrep. For bisulfite treatment, 400–500 ng of DNA was used for each column using EZ DNA Methylation-Gold Kit (Zymo; D5006). Two hundred microliters of water was used to elute DNA from each column. Real-time qPCR was performed in a 15 μl reaction containing 1 μl of the eluant using iQSYBRGreen (Bio-Rad, Hercules, CA, USA) in a Bio-Rad iCycler. The PCR primers (Supplementary Table S3) for methylated DNA were used at a final concentration of 0.3 μm in each PCR reaction. PCR was performed by initial denaturation at 95 °C for 5 min, followed by 40 cycles of 30 s at 95 °C, 30 s at 60 °C and 30 s at 72 °C. Specificity was verified by melting curve analysis and agarose gel electrophoresis. The threshold cycle (Ct) values of each sample were used in the post-PCR data analysis. Albumin DNA was used as loading controls for all QMSP normalization.

### Methyl-MiniSeq library construction

Libraries were prepared from 200 to 500 ng of genomic DNA digested with 60 units of TaqαI and 30 units of MspI (NEB) sequentially and then extracted with Zymo Research (ZR) DNA Clean & Concentrator-5 kit (Cat#: D4003). Fragments were ligated to preannealed adapters containing 5′-methyl-cytosine instead of cytosine according to Illumina's specified guidelines (www.illumina.com). Adaptor-ligated fragments of 150–250 and 250–350 bp in size were recovered from a 2.5% NuSieve 1:1 agarose gel (Zymoclean Gel DNA Recovery Kit, ZR Cat#: D4001). The fragments were then bisulfite-treated using the EZ DNA Methylation-Lightning Kit (ZR, Cat#: D5020). Preparative-scale PCR was performed and the resulting products were purified (DNA Clean & Concentrator-ZR, Cat#D4005) for sequencing on an Illumina HiSeq.

### Methyl-MiniSeq sequence alignments and data analysis

Sequence reads from bisulfite-treated EpiQuest libraries were identified using standard Illumina base-calling software and then analyzed using a Zymo Research proprietary analysis pipeline, which is written in Python and used Bismark (http://www.bioinformatics.babraham.ac.uk/projects/bismark/) to perform the alignment. Index files were constructed using the *bismark_genome_preparation* command and the entire reference genome. The *non_directional* parameter was applied while running Bismark. All other parameters were set to default. Filled-in nucleotides were trimmed off when doing methylation calling. The methylation level of each sampled cytosine was estimated as the number of reads reporting a C, divided by the total number of reads reporting a C or T. Fisher's exact test or *t*-test was performed for each CpG site which has at least five reads coverage, and promoter, gene body and CpG island annotations were added for each CpG included in the comparison.

### Cell viability and apoptosis

ARK2 and MCF-7 cells were seeded in 96-well plates at a density of 3 × 10^3^/well the night before metformin addition. Cell viability and caspase-3/7 activity were measured 48 h post metformin treatment using the CellTiterBlue Cell Viability kit (Promega, Madison, WI, USA) and the Apo-ONE Homogeneous Caspase-3/7 Assay kit (Promega), respectively, according to the manufacturer's protocols.

### Patients

Study approval was obtained from the Institutional Review Board of Chiba University. Five patients with endometrioid adenocarcinoma were recruited to the study. All patients signed an informed consent form according to the institutional guidelines. Eligibility criteria included an Eastern Cooperative Oncology Group performance status of 0 to 1 and normal renal, liver and cardiac function. Exclusion criteria were as follows: (1) type 2 diabetes requiring medication; (2) history of metformin use; (3) an abnormal blood coagulation profile and/or a history of thromboembolism; and (4) the presence of mental or life-threatening illnesses. All patients were obese with impaired glucose tolerance, but were not diabetic. Detailed characteristics of the patients are shown in Supplementary Table S1.

Metformin (initial dose, 750 mg/day; increased weekly up to 1500 or 2250 mg/day) was administered for 3–12 weeks until the day of scheduled surgery. Patients were not treated with any hormones such as progestin. Tissue specimens were obtained via endometrial curettage at the time of initial diagnosis (before treatment) and hysterectomy (after treatment).

### Tissue collection and analyses

For RNA extraction, tissue samples collected at surgery were snap frozen in liquid nitrogen and stored at −80 °C until RNA extraction. RNA was extracted from tissues using Trizol reagent (Life Technologies, Grand Island, NY, USA). cDNA was synthesized from 3 to 5 μg RNA using oligo-deoxythymidine 12–18 (catalog item 18418-012; Invitrogen) and SuperScript II reverse transcriptase (catalog item 18064-014; Invitrogen) in a 20 μl reaction volume according to the manufacturer's instructions. Real-time qPCR was performed as described above using β-tubulin mRNA as a loading control.

For immunohistochemical staining of Ki-67, 3-μm-thick sections were briefly microwaved in 10 mm of citrate buffer (pH 6.0) and immunostained for Ki-67. The Envision FLEX system (K8000; Dako, Carpinteria, CA, USA) was used to observe the immunostaining using an Autostainer S3400 (Dako). The primary antibody was incubated at room temperature for 60 min at a dilution of 1:100. The secondary antibody (Envision FLEX/HRP; Dako) was incubated at room temperature for 60 min, and 3,3′-diaminobenzidine tetrahydrochloride (Dako) was used as a chromogen. The samples were then counterstained with hematoxylin. Labeling indices for Ki-67 was presented as the percentage of immunoreactive nuclei of 500 tumor cells.

For AMPK western blot analysis, frozen tissue samples were thawed on ice, homogenized using a TissueRuptor (Qiagen) and lysed in Complete-M lysis buffer (Roche Applied Science, Tokyo, Japan) containing Halt phosphatase inhibitor cocktail (Thermo Fisher Scientific Inc., Wayne, MI, USA). Lysates (10 mg of protein) were resolved by 10% sodium dodecyl sulfate-polyacrylamide gel electrophoresis and transferred to nitrocellulose membranes (GE Healthcare Japan, Tokyo, Japan). The primary antibodies were diluted (1:1000 for phospho-AMPKa, AMPKa; and 1:5000 for β-actin) and incubated overnight at 4 °C. The secondary antibody (enhanced chemiluminescence horseradish peroxidase-conjugated anti-rabbit immunoglobulin G and anti-mouse immunoglobulin G; GE Healthcare) was incubated at room temperature for 60 min. Signals were detected using the ECL Select Western Blotting Detection Kit (GE Healthcare). Signal intensity was quantified using a densitometer (CS Analyzer version 3.0 software; ATTO, Tokyo, Japan) and normalized to β-actin levels.

For QMSP analysis, genomic DNA was extracted from frozen tissue samples using the Quick gDNA MicroPrep kit. QMSP was performed as described for ARK2 cells.

### Statistical analysis

All data (unless otherwise indicated) are presented as mean±s.d. All experiments were performed in triplicate and repeated at least three times. Statistical analyses were performed using the Statistical Package for the Social Science (SPSS) computer software version 17.0 (IBM SPSS Statistics, Chicago, IL, USA). The Student's *t*-test or the Mann–Whitney *U*-test, or the Wilcoxon signed-rank test were used to compare differences between quantitative variables when appropriate. The Fisher's exact text was used when appropriate, for comparing categorical variables (contingency tables). *P-*values at 0.05 or smaller (two-sided) were considered statistically significant.
