# Supplementary material for: Metformin alters DNA methylation genome-wide via the H19/SAHH axis
Source: Oncogene. 2016 Oct 24;36(17):2345–54. doi: 10.1038/onc.2016.391 (PMC5415944; doi:10.1038/onc.2016.391)
Supplement: Supplementary Information [file onc2016391x1.pdf]

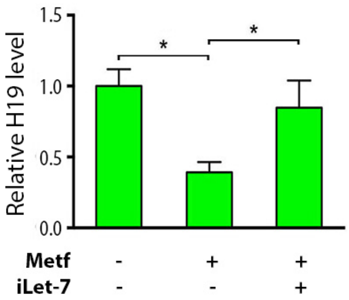

Supplementary Figure 1. Let-7 mediates metformin-induced H19 decrease. ARK2 cells were transfected with Let-7 inhibitor iLet-7 (iLet-7 +) or miRNA inhibitor control (iLet-7 -). The next day, cells were treated with metformin (Metf +) or water control (Metf -) and RNA was extracted 24 h later. RT-qPCR was carried out to determine H19 levels. Numbers are mean ± SD (n=3). *, $p < 0.05$.

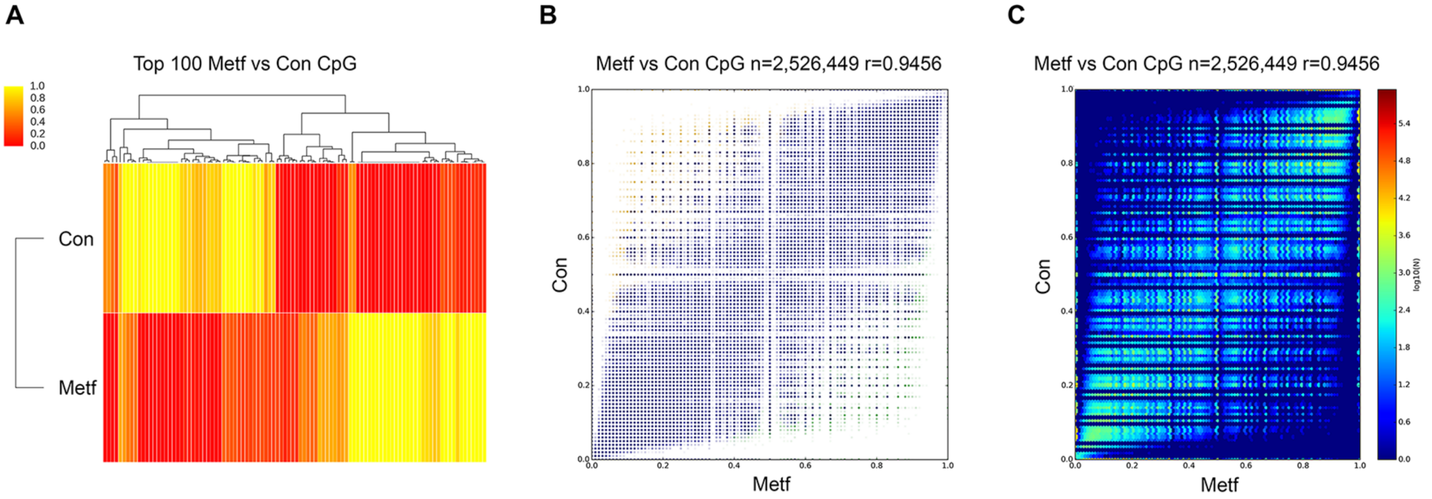

Supplementary Figure 2. (A) Hierarchical Clustering. Samples were grouped based on similarity for the top 100 differentially methylated CpG sites covered in the assay. Yellow represents high levels of DNA methylation, and red represents low levels of DNA methylation. (**B**) Pairwise Scatter Plot. Scatter plots depict correlation between two samples. For replicates, one would expect to see a straight "line" with slope=1, demonstrating perfect 1:1 correlation. Blue dots show sites with statistically insignificant differences between samples, significant difference sites are colored yellow and green. (**C**) Scatter Plot similar to **B** but using color to indicate the density of the CpG sites.

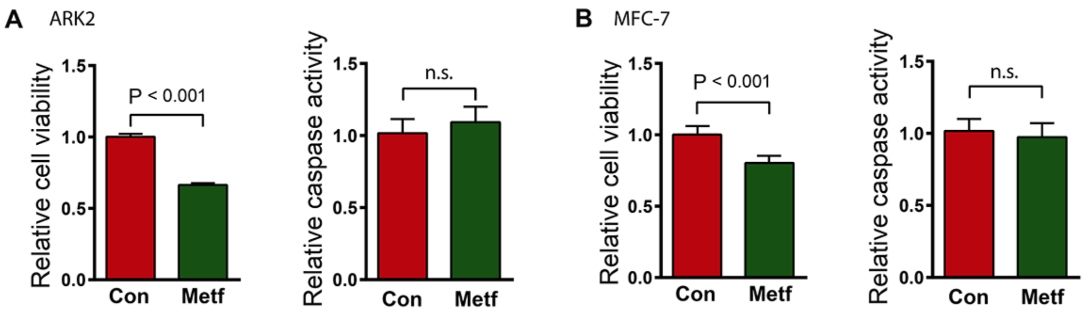

Supplementary Figure 3. Cell viability and apoptosis. ARK2 (**A**) and MCF-7 (**B**) cells were treated with Metformin or control. Forty-eight hours later, cell viability (left panels) and caspas 3/7 activity (right panels) were evaluated. The relative activities are presented with those of the Con group arbitrarily set as 1.

Supplementary Table 1. Characteristics of Patients

Patient ID	Age	Pathology	Stage	Height (m)	Weight (Kg)	BMI	HOMA-R	OGTT 120 min (mg/dl)	Metfomin (mg/day)	Treatment (wks)
Case 1	39	Endometrioid (G1)	IA	1.59	80.1	31.7	5.63	177	2250	7
Case 2	28	Endometrioid (G1)	IA	1.6	93.6	36.6	2.57	144	2250	7
Case 3	49	Endometrioid (G1)	IIA	1.56	80	32.9	7.51	198	1500	7
Case 4	30	Endometrioid (G1)	IA	1.6	90	35.1	8.96	192	1500	12
Case 5	43	Endometrioid (G1)	IA	1.61	131	50.5	4.55	141	1500	3

Supplementary Table 2. Real-time PCR primer sequences

Gene	Forward primer	Reserve Primer
H19	5'-ACTCAGGAATCGGCTCTGGAA-3'	5'-CTGCTGTTCCGATGGTGTCTT-3'
Gapdh	5'-CTTTGTCAAGCTCATTTCCTGG-3'	5'-TCTTCCTCTTGTGCTCTTGC-3'
Dnmt3b	5'-TTGGAATAGGGGACCTCGTGTG-3'	5'-AGAGACCTCGGAGAACTTGCCATC-3'
RPL22	5'-GGAGCAAGAGCAAGATCACC-3'	5'-TCCTCCTCTTCTTCGTCCTG-3'
TUBB	5'-CGTGTTCGGCCAGAGTGGTGC-3'	5'-GGGTGAGGGCATGACGCTGAA-3'
ACTIN	5'-ATCAAGATCATTGCTCCTCCTGAG-3'	5'-CTGCTTGCTGATCCACATCTG-3'

Supplementary Table 3. QMSP PCR primer sequences

Gene	Forward primer	Reserve Primer
DMRTA2	5'-TGTTCGGTGCGTTTTTTTGCG-3'	5'-AAACGATACAACCGAACCGACG-3'
KSNG2	5'-TTTTTATCGGTCGGTGGGGTTC-3'	5'-CGAACGAAACTTTCACAAATCGACG-3'
PSMD10	5'-GGGTTTTTGATTTGTAGTGGTAGGC-3'	5'-AAAAAATAACCACAATCACCGATTCG-3'
TRA2A	5'-TGGATTTAAGCGATTTGTTCGTTTC-3'	5'-ATATATACACGATTCCAACCGAACG-3'
H19	5'-GGGAGTTATATTACGTTTTCGTATCG-3'	5'-TACTATACTCATCACGCGAATAAACG-3'
Albumin	5'-GTATGCCTGAGCCCCAAAGT-3'	5'-CCTTGGGCTTGTGTTTCACG-3'
